# How do personality traits manifest in daily life of older adults?

**DOI:** 10.1007/s10433-020-00598-z

**Published:** 2021-02-17

**Authors:** Stefanie Lindner, Damaris Aschwanden, Johannes Zimmermann, Mathias Allemand

**Affiliations:** 1 Department of Psychology and University Research Priority Program Dynamics of Healthy Aging, University of Zurich, Andreasstrasse 15, CH-8050 Zurich, Switzerland; 2 Department of Geriatrics, Florida State University, Tallahassee, USA; 3 Department of Psychology, University of Kassel, Kassel, Germany

**Keywords:** Personality trait manifestations, Traits, Experiences, Behaviors, Older adults, Ambulatory assessment

## Abstract

The present study examined how personality traits manifest in daily life of older adults and distinguished between the manifestations of experiences and behaviors. We used data from an ambulatory assessment study over 10 days with assessments of trait-related experiences and behaviors obtained from 136 older adults aged between 60 and 91 years (41.2% male; *M* = 70.45 years). Multilevel models revealed that on average, 61.2% of variance in trait-related experiences and 39.6% of variance in behaviors were due to consistent differences between persons. Older adults were rather variable and diverse in their trait manifestations, while they also showed relative stability in trait manifestations. Across older age, some age effects for trait manifestations were found. Moreover, within-person variation of experiences and behaviors showed, with one exception, joint fluctuations in daily life. The findings portray a nuanced picture of trait manifestations in older adulthood. The findings complement the literature on within-person variability in older adulthood and might encourage further studies from a within-person perspective to better understand how older adults navigate through daily life.

**Supplementary Information:**

The online version contains supplementary material available at (10.1007/s10433-020-00598-z).

## Introduction

Aging researchers have noted the importance of studying psychological aging processes in daily life of older adults. For example, one line of research examined cognitive processes in naturalistic settings and found that greater variability in daily activities was related to greater variability in daily cognitive performance (Bielak et al. 2019). Another line of research investigated daily stressors and found that higher stressor diversity was associated with better well-being across adulthood (Koffer et al. 2016). Furthermore, research examined social processes in daily life and showed that the intraindividual variability of interaction frequency with peripheral partners was lower among older adults as compared to younger adults (Zhaoyang et al. 2018). Another important domain of psychological functioning is personality. However, little is known about *how* personality traits manifest or express in daily life of older adults.

The primary goal of the present study was to examine the manifestations of personality trait-related experiences and behaviors in late life as individuals go about their daily lives. We aimed to complement the current literature on personality development by applying a within-person perspective on trait manifestations. Variability and diversity in trait manifestations, if true, may reflect mechanisms and moderators that are important to understand aging outcomes. For example, variability in open experiences may reflect an effective use of cognitive engagement depending on the activity, which may bestow an advantage in cognitive aging. Moreover, diversity in extraverted behaviors may allow individuals to choose among context-appropriate responses, which then might contribute to enhanced well-being in social interactions. Building some ground for future work, we aimed to investigate the basic characteristics and links of trait-related experiences and behaviors in a sample of adults aged between 60 and 91 years using data from an ambulatory assessment study. There are few ambulatory assessment studies available to date to test the extent to which trait manifestations varies within older age. We attempted to fill this gap by addressing three research questions: First, how stable, variable, and diverse are trait-related experiences and behaviors in daily life of older adults? Second, how are age and personality traits related to trait-related experiences and behaviors? Third, do trait-related experiences and behaviors fluctuate in concert?

### Conceptualizing personality trait manifestations

Personality traits are typically defined as relatively enduring patterns of experiences (thoughts, feelings) and behaviors (Roberts and Jackson 2008). Despite the relatively enduring nature of traits, their manifestations or expressions in daily life may show variability and diversity over time. The present work focuses explicitly on the everyday manifestations of the Big Five traits, defined as trait-related experiences (e.g., being calm, emotionally stable [neuroticism]) and behaviors (e.g., finishing a task on time [conscientiousness]) at a particular time (Roberts and Jackson 2008).

A focus on short-term personality variability and diversity is crucial from an aging perspective. To better understand aging processes in personality, it is important to complement research efforts on long-term development (e.g., over years and decades) with aging research on short-term processes in daily life (e.g., over days; Diehl et al. 2015; Nesselroade 1991). Such an integrative perspective is emphasized in several theories on personality (Fleeson and Jayawickreme 2015; Hooker and McAdams 2003; Wrzus and Roberts 2017). For example, the descriptive part of the Whole Trait Theory (WTT; Fleeson and Jayawickreme 2015; Jayawickreme et al. 2019) provides a unique account on how personality traits translate into daily experiences and behaviors. WTT argues that personality traits represent aggregates of experiences and behaviors that can be characterized as a density distribution, whose mean corresponds to the individual’s trait level. Deviations from the mean refer to variability in experiences and behaviors, indicating that individuals exhibit a range of personality trait manifestations in daily life but tend to vary around their individual trait level.

The explanatory part of the WTT argues that trait manifestations are a function of situational and individual influences (Fleeson and Jayawickreme 2015). On the one hand, individuals engage in a variety of daily situations that influence behaviors and cause variability of experiences and behaviors at a particular time (Fleeson 2007; Sherman et al. 2015). For example, having multiple opportunities to engage in intellectually challenging experiences may result in more open behaviors, but not everyone will take advantage of these opportunities. On the other hand, individuals differ in their interpretations of and reactions to daily situations. Several individual processes might influence the daily manifestations of traits, such as interpretative, motivational, and stability-inducing processes (Fleeson and Jayawickreme 2015). For instance, the interpretation of a situation as favorable toward openness may lead to more open experiences and more diverse open behaviors. The present study refers to the descriptive part of the WTT.

### Describing personality trait manifestations

There are several statistical approaches to describe how experiences and behaviors manifest in everyday life (Wright and Zimmermann 2019). One approach is to use the intraindividual mean (i*M*). The i*M* is the average score of a repeatedly assessed variable within an individual across a given time period. A second approach refers to deviations from the i*M*, called intraindividual variability (i*SD*). The i*SD* describes the tendency toward fluctuations of trait manifestations within an individual over time. A third approach refers to diversity and can be defined as the tendency to show a variety of behaviors across a given time period. Unlike i*M*, diversity assesses the range and uniformity of displayed behaviors and thus indicates how diverse a behavior repertoire is. Individuals may differ in their manifestations of more or less diverse behaviors. Whereas aging researchers have recognized the need to examine diversity in domains such as stress, social interactions, and daily activities (Fingerman et al. 2019; Jackson et al. 2019; Koffer et al. 2016), this approach has not yet been applied to personality.

Prior research with younger samples showed that individuals deviate substantially from their i*M* in trait manifestations (Fleeson and Gallagher 2009; Geukes et al. 2017). Even though individuals showed i*SD*, individual differences in these deviations tended to be relatively stable. That is, individuals were relative consistent in their amount of i*SD* around their i*M* (Fleeson 2007; Noftle and Fleeson 2010). Taken together, these findings suggest that trait manifestations are characterized by both stable and variable aspects.

### Personality traits and their manifestations

Previous studies with younger samples examined the associations between personality traits and the average of repeated assessments of trait manifestations (Fleeson and Gallagher 2009; Rauthmann et al. 2019). This research found that personality traits and their corresponding trait manifestations are moderately correlated ranging from .18 to .56. Noteworthy, personality traits differ in their composition of experiences and behaviors, with some traits being stronger represented by experiences (i.e., neuroticism, openness), behaviors (i.e., extraversion, conscientiousness), or a mix of experiences and behaviors (i.e., agreeableness) (Pytlik Zillig et al. 2002; Wilt and Revelle 2015). One caveat of previous studies is that they typically assessed trait manifestations using adjective-based measures that do not explicitly distinguish between experiences and behaviors (e.g., “energetic” for extraversion; “warm” for agreeableness; Fleeson 2007). An alternative but rarely applied method is to use checklists such as the Daily Behavior Checklist (DBQ; Church et al. 2008) to assess behavioral manifestations of traits (e.g., “gave in to a bad habit when I was nervous” [neuroticism]; “went out to socialize” [extraversion]; Table S1). Previous work using the DBQ found moderate correlations between traits and corresponding behaviors in younger samples (with the lowest effect of *β* = .13 for disagreeableness [reversed to measure agreeableness] and highest effects of *β* = .27 for neuroticism [reversed to measure emotional stability]) (Hudson and Roberts 2014). However, it is still unclear how personality traits translate into daily experiences versus behaviors, and whether and to what degree experiences and behaviors are interrelated in daily life. Traits are typically understood as composites of experiences and behaviors (e.g., Roberts and Jackson 2008). This suggests that what an individual thinks and feels (experiences) is simultaneously reflected in their behaviors. However, there may be situations in which experiences could be detached from behaviors. For example, an individual may think in an open-minded way, but not act in an open-minded way because the situation does not provide an opportunity for it or the individual is not motivated. Measuring experiences and behaviors separately cannot only provide insight into how personality components are translated into daily life, but also help to understand whether and how manifestations of experiences and behaviors of the same traits are actually connected within individuals over repeated assessments in daily life (Wilt and Revelle 2015).

### Personality trait manifestations in older age

An increasing number of studies have demonstrated that personality traits are malleable and show developmental change in older age (Chapman et al. 2007; Kandler et al. 2015). However, little is known about how traits manifest in daily life of older adults. There are at least two possibilities about how traits are manifested in older adults. One possibility is that older adults show less variability and diversity in their trait manifestations. This means, older adults might hardly fluctuate from low to high scores in, for example, neurotic experiences (i.e., less variability) or show a low number of different neurotic behaviors (i.e., less diversity). Older people may experience more age-related challenges such as cognitive and physical declines, which may constrain their engagement in or reactions to daily situations (Schaie and Willis 2015). For example, compromised health (e.g., hearing impairment) may reduce the capacity to participate in social activities, which could restrict the repertoire of extraverted behaviors (Berg and Johansson 2014). Due to restricted social activities, trait manifestations are likely to become more regular and uniform over time (Noftle and Gust 2019). Furthermore, older adults tend to prefer situations that match their personality traits, resulting in lower variability of experiences and diversity in behaviors (Wrzus et al. 2016). Finally, the accumulation of habits can result in a routinized and less variable trait manifestations (Fleeson and Jayawickreme 2015).

Another possibility is that older adults increase in their variability and diversity in trait manifestations. That is, older adults may fluctuate from low to high scores over time in experiences (more variability) and show a high number of different behaviors (more diversity). For example, expectations about certain social roles are more open and less structured in older adulthood compared to previous life phases (Freund et al. 2009), which may result in greater scope for variability in experiences or a broader repertoire of behaviors. Furthermore, with increasing age, more differentiated interpretations of situations may contribute to more nuanced and variable experiences as well as diverse behaviors tailored to these situations (Noftle and Gust 2019). Relatedly, more adaptive emotion regulation strategies may facilitate variation in trait manifestations and more context-appropriate responses (Schirda et al. 2016). Accumulation of habits could also result in a wider behavioral repertoire and thus more diverse manifestations with age.

While previous studies with older adults showed evidence for both stability and variability in the manifestations of other aspects of personality (Eizenman et al. 1997; Hooker et al. 2013), less is known about the manifestations of the Big Five traits. To the best of our knowledge, only Noftle and Fleeson (2010) examined trait manifestations in older adults (in comparison to middle-aged and younger adults) across 2 weeks. The results demonstrated a sizeable amount of stability in interindividual differences (split-half reliability) and intraindividual variability in trait manifestations for all age groups. While this work provides initial evidence for stability and i*SD* in the trait manifestations of older adults, it did not explicitly separate trait-related experiences and behaviors. Examining varying and contextualized behaviors and not only experiences would contribute to a better understanding of how trait content manifests in daily life. Finally, it is an open question how diversely older adults behave on a day-to-day basis. Demonstrating diversity in trait manifestations would suggest that personality manifestations of older adults are not characterized by rigidity and stagnation but rather reflect dynamic aging processes (Ram and Gerstorf 2009).

### The present study

The present study was centered around three research questions: First, how do experiences and behaviors manifest in daily life of older adults? Based on our theorizing and available research (Noftle and Fleeson 2010), we expected that trait manifestations are characterized by both stability and variability. To describe experiences, we focused on the i*M* and i*SD*. To describe behaviors, we focused on i*M*, i*SD*, and diversity. Furthermore, we investigated the week-to-week stability of individual differences in i*M* and i*SD* of experiences and behaviors. Based on previous research (Fleeson 2007; Noftle and Fleeson 2010), we expected higher stability coefficients for the i*M* than for the i*SD* of experiences and behaviors.

Second, how are age and personality traits related with experiences and behaviors in older adults? Based on previous work with younger adults (Fleeson and Gallagher 2009; Rauthmann et al. 2019), we expected that traits are positively associated with corresponding experiences and behaviors. Because the DBQ measures disagreeable behaviors, we expected a negative association between trait agreeableness and disagreeable behaviors. Extending previous work, we did not only investigate associations between traits and experiences but also between traits and behaviors.

Third, how are experiences and behaviors associated in older adults? We examined whether repeatedly assessed experiences and behaviors co-jointly fluctuate in daily life. We expected that experiences are positively related to corresponding behaviors in daily life. For the relationship between agreeable experiences and disagreeable behaviors, we expected a negative association. We examined the associations between experiences and behaviors both between individuals and within individuals.

## Methods

### Participants

Participants came from the RHYTHM study (Realizing Healthy Years Through Health Maintenance) in Switzerland^1^. Older adults aged 60+ years were recruited via advertisements in a newspaper and research participant pools. The sample included 136 participants (41.2% male) and ranged in age from 60 to 91 years (*M* = 70.45, SD = 6.27). None of the participants showed signs of cognitive impairment (Mini Mental State Examination scores > 24; Folstein et al. 1975) or depression (General Depression Scale scores < 18; Hautzinger and Bailer 1993). On average, participants showed relatively good subjective health (*M* = 3.76, SD = 0.69), as measured with a single item on a scale from 1 (*poor*) to 5 (*excellent*). The marital status of the sample was 7.4% single, 46.7% married, 2.2% separated, 30.4% divorced, and 13.3% widowed. Of the participants, 3.7% attended secondary school with lower school track, 15.4% attended secondary school with higher school track, 3.7% attended secondary school with the Matura graduation, 25.7% attended a university of applied sciences, 20.6% attended university, and 30.9% reported to have another educational background (e.g., vocational training). Education was coded as a dummy variable.

### Study design and procedure

The ambulatory assessment study was approved by the Ethics Committee for psychological and related research of the University of Zurich. Written informed consent was obtained from all participants after a detailed explanation of the study. The study included a pre-daily assessment (Day 1) and ambulatory assessments (from Days 2 to 11). On Day 1, participants came to the laboratory for a screening session, completed a series of questionnaires, received instructions on the ambulatory assessment sampling period and were equipped with a smartphone with MovisensXS software (version 4474; movisens GmbH 2016). The ambulatory assessment phase lasted 10 days. On each day, three assessments were randomly timed within a fixed time period (morning: between 08:00 and 11:00 am.; afternoon: between 01:00 and 04:00 pm.; evening: between 06:00 and 09:00 pm.). Experiences were assessed in the morning and afternoon, and behaviors were assessed retrospectively in the evening only. Participants were paid 150 Swiss Francs (approx. USD 150) for their participation. The compliance of the participants was high, with 96% completed measurement occasions for experiences and 92% for behaviors.

### Dispositional measures

#### Big Five personality traits

Traits were measured using the Big Five Inventory (BFI; John and Srivastava 1999) on Day 1. Each trait was assessed by 8–10 items (e.g., “emotionally stable, not easily upset” inverted for neuroticism). The scale ranged from 0 (*strongly disagree*) to 6 (*strongly agree*). Omega hierarchical estimates ranged from .45 (openness) to .66 (neuroticism). Except for the omega of openness, the reliabilities were acceptable to good.

### Daily measures

#### Big Five personality experiences

Experiences were assessed with the Ten Item Personality Inventory (TIPI; Gosling et al. 2003) from Day 2 until Day 11. Twice per day, participants rated the degree to which each item described them during the past 2 h. Each Big Five trait was measured with two items (e.g., “anxious and easily upset”, “calm and relaxed” for neuroticism). The scale ranged from 0 (*strongly disagree*) to 6 (*strongly agree*). Estimates of the intraindividual reliability of change (cf. Shrout and Lane 2012) ranged from .52 (conscientious experiences) to .84 (open experiences), suggesting that the two-item measures for each Big Five trait assessed intraindividual variability reliably (fair to substantial).

#### Big Five personality behaviors

Behaviors were assessed with the Daily Behavior Checklist (DBQ; Church et al. 2008) from Day 2 until Day 11. Every evening, trait-related behaviors were assessed with 50 items (Table S1). Participants checked “yes” (1) or “no” (0) to indicate whether they had shown this behavior that day. We slightly modified two items for the use with older adults^2^. Note that disagreeableness was measured (not agreeableness). Each Big Five trait was assessed with 10 items. The daily sum score for each trait could range from 0 to 10. Estimates of the intraindividual reliability of change ranged from .40 (disagreeable and conscientious behaviors) to .83 (extraverted behaviors), suggesting that the ten-item measures for each Big Five trait assessed intraindividual variability reliably (fair to substantial).

### Covariates

To evaluate the extent to which the associations between personality traits, experiences, and behaviors are robust to potential confounds, we controlled for age, gender (0 = *female*, 1 = *male*), subjective health, and education (Chapman et al. 2007; Donnellan and Lucas 2008).

### Analysis strategy

For the first research question, we investigated between-person differences in the manifestations of experiences and behaviors relative to within-person variations using multilevel modeling (MLM; Bolger and Laurenceau 2013). We estimated unconditional random-intercept-only models without independent variables to calculate the intraclass correlation coefficients (ICCs). We computed i*M*, i*SD*, and their stability across the study period to describe manifestations of experiences and behaviors. To test for stability, we computed split-half reliability by dividing each individual’s data into equal time periods^3^ (Fleeson 2007; Noftle and Fleeson 2010). For each time period, i*M* and i*SD* are calculated and correlated. Following other researchers (e.g., Zhaoyang et al. 2018), we used Shannon’s (1948) entropy index to assess diversity of behaviors (see Appendix S1 for details).

For the second research question, we computed bivariate Pearson correlations to examine how age and baseline trait levels were associated with trait manifestations in daily life (i.e., i*M*, i*SD*, and diversity). Because the DBQ measures disagreeable behaviors, we expected a negative association between trait agreeableness and disagreeable behaviors. Additionally, we tested the robustness of these associations by controlling for potential effects of age, gender, subjective health, and education using partial correlations.

For the third research question, we used MLM to examine within-person associations between trait-related experiences and behaviors. The data exhibited a nested structure: Daily observations (Level 1) were nested within participants (Level 2). We performed random-intercept-random-slope models with experiences as dependent variables and behaviors as independent variables. The data structure consisted of 20 measurement points of experiences per person. We included a between-person version and a within-person version of the independent variables to control for the between-person effects and to truly examine the within-person variation (Bolger and Laurenceau 2013). We included time as a fixed and random effect to control for potential reactivity effects and individual differences in change. We applied bootstrapping procedure (with 1000 bootstrap samples) to derive bias-corrected 95% confidence intervals for each parameter. Additionally, we tested the robustness of the associations by controlling for age, gender, subjective health, and education as between-person covariates.

## Results

### Describing trait-related experiences and behaviors

Descriptive statistics of experiences and behaviors are shown in Tables 1 and 2 (zero-order correlations among the main study variables are shown in Table S2). The relative amount of between-person variance for experiences ranged from .54 (open experiences) to .67 (neurotic and conscientious experiences). The relative amount of between-person variance for behaviors was lower than for experiences and ranged between .31 (neurotic behaviors) and .55 (open behaviors), suggesting that there was more variation within persons in behaviors. Regarding average daily i*M,* i*SD*, and diversity scores, we found interindividual differences. Figure S1 illustrates a selection of individual trajectories of i*SD* in neurotic experiences. Figure S2 illustrates a selection of individual trajectories of daily behaviors across 10 days. In line with our expectation, week-to-week stability estimates were higher for both i*M* in experiences and behaviors than for i*SD* in experiences and behaviors (see Appendix S2 for more details).

**Table 1:** Descriptive statistics of trait-related experiences and variance decomposition

Experiences	ICC	Intraindividual mean (i*M*)				Intraindividual variability (i*SD*)				Stability (*r*)	
		*M*	*SD*	Min	Max	*M*	*SD*	Min	Max	i*M*_w1w2_	i*SD*_w1w2_
Neuroticism	.67	1.12	0.98	0	4.25	0.53	0.43	0	2.83	.96*	.69*
Extraversion	.63	3.47	1.17	1.25	5.90	0.80	0.39	0.21	2.48	.95*	.82*
Openness	.54	3.51	0.80	1.80	6.00	0.64	0.36	0	2.08	.93*	.67*
Agreeableness	.55	4.87	0.77	2.20	6.00	0.59	0.37	0	2.01	.91*	.54*
Conscientiousness	.67	4.88	0.91	1.50	6.00	0.53	0.36	0	2.36	.96*	.69*

*N* = 136 participants, observations = 2685 − 2687; ICC: intraclass correlation, i*M*_w1w2_: stability correlations of experiences for i*M* between Weeks 1 and 2, i*SD*_w1w2_: stability correlations of experiences for i*SD* between Weeks 1 and 2.

*p < .001

**Table 2:** Descriptive statistics of trait-related behaviors and variance decomposition

Behaviors	ICC	Intraindividual mean (i*M*)				Intraindividual variability (i*SD*)				Diversity				Stability (*r*)	
		*M*	*SD*	Min	Max	*M*	*SD*	Min	Max	*M*	*SD*	Min	Max	i*M*_w1w2_	i*SD*_w1w2_
Neuroticism	.31	1.23	0.56	0.10	3.80	0.66	0.42	0	1.89	0.93	0.64	0	2.08	.43**	.29**
Extraversion	.34	3.97	1.54	0	8.25	1.89	0.67	0	3.60	2.08	0.29	0	3.49	.64**	.34**
Openness	.55	2.87	1.43	0	7.00	1.16	0.44	0	2.31	1.88	0.36	0	3.04	.83**	.24**
Disagreeableness^a^	.32	0.70	0.74	0	3.60	0.84	0.64	0	3.15	1.68	1.23	0	4.82	.44**	.24**
Conscientiousness	.46	6.13	1.17	3.11	9.44	1.13	0.43	0	2.57	2.07	0.17	1.43	2.37	.74**	.33**

*N* = 136 participants, observations = 1252

ICC: intraclass correlation, i*M*_w1w2_: stability correlations of behaviors for i*M* between Weeks 1 and 2, i*SD*_w1w2_: stability correlations of behaviors for i*SD* between Weeks 1 and 2

^a^The daily behavior checklist assessed disagreeable instead of agreeable behaviors (Church et al. 2008)

*p < .01, ** *p* < .001

### Age, traits, and trait-related experiences

Correlational findings are shown in Table 3. These results suggest that higher age was associated with higher i*M* in neurotic experiences and more i*SD* in neurotic, extraverted, and open experiences. In line with our hypothesis, personality traits were positively associated with i*M* in corresponding experiences (*r* range =.41–.57), except for openness (*r* = − .16, *p* = .068). There were also some significant noncorresponding trait-related experiences associations. With respect to i*SD* in experiences, high trait neuroticism was related to more i*SD* in neurotic experiences, while high trait agreeableness was related to less i*SD* in agreeable experiences. Furthermore, we found some unexpected associations with respect to i*SD* in neurotic experiences. High trait scores in openness, agreeableness, and conscientiousness were associated with less i*SD* in neurotic experiences. Results of additional analyses with covariates (Table S3) were similar to results without covariates.

**Table 3:** Zero-order correlations between traits and trait-related experiences

Traits	Intraindividual mean (i*M)*					Intraindividual variability (i*SD*)				
	*N*	*E*	*O*	*A*	*C*	*N*	*E*	*O*	*A*	*C*
Neuroticism	.57***	− .38***	.00	− .33***	− .32***	.25***	.10***	.08***	.12***	.09***
Extraversion	− .23***	.50***	− .01	.32***	.20***	.03***	.10***	− .03***	.10***	.08***
Openness	− .34***	.14***	− **.16**	.23***	.31***	− .19***	.08***	− .03***	− .06***	− .06***
Agreeableness	− .37***	.30***	− .07	.41***	.28***	− .25***	− .18***	− .23***	− .25***	− .20***
Conscientiousness	− .37***	.26***	− .01	.32***	.55***	− .28***	− .13***	− .14***	− .10***	− .14***
Age	.21***	− 16	.02	.03***	− .12***	.25***	.30***	.09***	.07***	.06***

*N* = 136 participants; correlations between traits and the corresponding experiences are printed in bold face

*p < .05, **p < .01, ***p < .001

### Age, traits, and trait-related behaviors

The associations between age and trait-related behaviors are shown in Table 4. Age was not significantly related to any of the three descriptors i*M*, i*SD*, diversity. Correlational results supported the expected associations between traits and the corresponding i*M* of the behaviors for all traits, ranging from .20 (extraverted behaviors) to .40 (neurotic behaviors). Regarding i*SD* in behaviors, higher trait levels of neuroticism and openness were related to more i*SD* in corresponding behaviors, whereas higher trait levels of agreeableness were linked to less i*SD* in disagreeable behaviors. Compared to experiences, we found generally fewer significant associations between traits and noncorresponding behaviors. For example, high trait neuroticism was significantly related to more i*SD* in neurotic, disagreeable, and conscientious behaviors. High levels in trait neuroticism, extraversion, and openness were associated with more diverse corresponding behaviors in daily life. High agreeableness was associated with less diversity of neurotic behaviors. Results of additional analyses with covariates (Table S4) did not show substantial departures from the above reported results.

**Table 4:** Zero-order correlations between traits and trait-related behaviors

Traits	Intraindividual mean (i*M*)					Intraindividual variability (i*SD*)					Diversity				
	*N*	*E*	*O*	*D*^a^	*C*	*N*	*E*	*O*	*D*^a^	*C*	*N*	*E*	*O*	*D*^a^	*C*
N	.40***	− .13**	.04**	.27**	− .15	.36***	.04	.11	.33***	.21*	.35***	.02	.02	− .01	.08
E	− .07*	.20*	.19**	.12	.08	− .07	**.16**	.13	.07	− .06	− 12	.20*	.20*	.09	.06
O	− .07**	.17*	.26**	− .03	.16*	.00	.10	.27**	.12	.08	− .03	.16	.18*	.06	.14
A	− .24**	.21*	.06*	− .20**	.21**	− .18*	− .10	.07	− .29***	− 04	− .25**	− .03	− .06	− **.07**	− .01
C	− .26**	.16*	.10*	.10	.32***	− .13	− .05	.06	− .12	− **.16**	− .12	− .03	− .02	.02	− **.03**
Age	− .02*	− .12**	.10*	.12	− .12	.03	− .07	.00	.05	− .10	.01	− .09	− .01	.10	− .16

*N* = 136 participants; correlations between traits and corresponding behaviors are printed in bold face

^a^The daily behavior checklist assessed disagreeable instead of agreeable behaviors (Church et al. 2008)

*p < .05, **p < .01, ***p < .001

### Trait-related experiences and behaviors

Results of the MLM analyses are shown in Table 5. As expected, neurotic, extraverted, disagreeable, and conscientious behaviors were associated with the corresponding experiences at the between-person and within-person level. For example, more daily neurotic behaviors were associated with more daily neurotic experiences at the between-person level (*b* = .68, *SE* = .14, *p* < .001, 95 %CI [.41, .95]). This suggests that those people with higher scores in neurotic behaviors across 10 days also showed higher scores in daily neurotic experiences. At the within-person level, days of more neurotic behaviors were related to more neurotic experiences on the same days (*b* = .09, *SE* = .02, *p* < .001, 95 %CI [.05, .13]). For openness, we did not find significant associations between experiences and behaviors at the between-person and within-person level, suggesting that open behaviors were not related with open experiences neither across the 10 days nor at the daily level. Finally, including covariates in the MLM models (Table S5) produced similar findings.

**Table 5:** Fixed effects of multilevel modeling of trait-related behaviors on corresponding experiences, without control variables

Experiences										
Behavior	Neuroticism		Extraversion		Openness		Agreeableness^a^		Conscientiousness	
	Estimate (SE)	CI 95 %	Estimate (SE)	CI 95 %	Estimate (SE)	CI 95 %	Estimate (SE)	CI 95 %	Estimate (SE)	CI 95 %
Intercept	.33 (.20)	[.00, .68]	2.24*** (.26)	[1.75, 2.78]	3.66*** (.15)	[3.41, 4.01]	4.99*** (.09)	[4.80, 5.17]	3.01*** (.38)	[2.28, 3.85]
Between	.68*** (.14)	[.41, .97]	.30*** (.06)	[.17, .41]	− .04 (.05)	[− .16, .03]	− .30* (.13)	[− .55, -.04]	.30*** (.06)	[.16, .42]
Within	.09*** (.02)	[.06, .13]	.04*** (.01)	[.03, .06]	.01 (.01)	[− .01, .03]	− .07** (.02)	[− .12, − .03]	.04*** (.01)	[.02, .05]
Time	.00* (.00)	[− .01, .00]	.01 (.01)	[.00, .01]	.00 (.00)	[− .01, .00]	.00 (.00)	[.00, .01]	.00* (.00)	[.00, .01]

*N* = 136 participants, observations = 2493; estimates are unstandardized multilevel regression coefficients with standard errors in parentheses; bias-corrected 95% confidence intervals based on the bootstrap method (with 1000 bootstrap samples)

Between: between-person version of the independent variable; within: within-person version of the independent variable; CI: confidence interval

aThe independent variable was disagreeable behaviors as assessed with the Daily Behavior Checklist (Church et al. 2008)

*p < .05, **p < .01, ***p < .001

## Discussion

The goal of this study was to contribute to the aging literature by describing trait manifestations of older adults in different ways. Overall, the results revealed a nuanced picture of personality stability, variability, and diversity in daily life. On one hand, older adults showed a relatively high week-to-week stability in i*M* and i*SD*, suggesting that individual differences in trait manifestations were maintained across the 10 days. This is in line with previous ambulatory assessment research (Fleeson 2007; Noftle and Fleeson 2010). It is important to note that experiences and behaviors differed in their relative stability, with behaviors showing weaker week-to-week stability than experiences. Likewise, for behaviors, we found more variation within individuals than between individuals. These findings are interesting and novel per se, given this is one of the first studies distinguishing between experiences and behaviors. Multiple determinants, such as goals and situational factors influence experiences and behaviors (McCabe and Fleeson 2016). It is likely that experiences and behaviors were differently affected by these determinants, leading to differences in daily variation. However, we cannot exclude that these differences were partly driven by differences in the specificity of the measures. Whereas the adjective-based TIPI assessed experiences on a rather global level, the DBQ assessed behaviors in a narrower and more contextualized way, which could have led to more variation. Future studies that measure experiences and behaviors on the same specificity level are needed to exclude this possibility.

On the other hand, older adults showed variability and diversity in experiences and behaviors. Our i*SD*-findings are consistent with previous studies (Fleeson and Gallagher 2009; Geukes et al. 2017) and extend previous research by providing further support in a sample of older adults. Personality variability might reflect flexible adaptations to challenges and opportunities in daily situations (Fleeson 2007; Kashdan and Rottenberg 2010). For example, the role of a grandparent may increase variability and diversity of extraverted experiences and behaviors. Moreover, behavioral diversity might be beneficial for various aging outcomes. For instance, a diverse repertoire of extraverted behaviors allows individuals to choose among different context-appropriate responses that may facilitate social engagement, which in turn is associated with increased well-being (Fingerman et al. 2019). Similarly, diversity in open behaviors might facilitate the engagement in cognitive activities, which in turn fosters cognitive abilities (Jackson et al. 2019). Future research should further investigate the role of diversity in personality manifestations in promoting aging outcomes.

Age was positively associated with neurotic experiences, suggesting that with increasing age, participants felt more anxious or less calm across the 10 days of the study. This result complements developmental research showing increases in neuroticism in late life (Kandler et al. 2015). Moreover, age was associated with more daily variability in neurotic, extraverted, and open experiences. It is possible that daily challenges, evoked by health constraints and functional impairments in old age (Smith et al. 2002), might have produced more fluctuations in neurotic experiences. More variability in extraverted experiences in old age might be explained by more variability in the frequency of social interactions (Zhaoyang et al. 2018). Variability in open experiences might reflect flexible responses to cognitive constraints in daily life (Bielak et al. 2019).

With some exceptions, personality traits were significantly related to the i*M* in the corresponding experiences and behaviors. These findings are in line with previously reported convergent correlations between baseline trait level and trait manifestations over multiple time scales (Fleeson and Gallagher 2009; Rauthmann et al. 2019). Our results extend previous work by distinguishing between experiences and behaviors. Interestingly, trait openness was not related to i*M* in open experiences but with i*M* in open behaviors. A possible explanation is that the retrospective self-concept of openness depicts daily behavioral expressions of openness better than open experiences in older adults. Alternatively, the nonsignificant relationship can also be the result of an imbalance of cognitive and affective items between the two measures of openness (BFI and TIPI), as the BFI items for openness are mainly cognitive (Pytlik Zillig et al. 2002) which may not correspond to the daily manifestations of openness assessed with the TIPI ("open to new experiences, complex" and "conventional, uncreative" (reversed)). Future studies are needed to clarify how the cognitive, affective, and behavioral components of measures are reflected in trait manifestations in daily life.

Personality traits were differently linked to i*SD* in trait manifestations, supporting previous findings (Fleeson and Gallagher 2009). For instance, a higher trait level in neuroticism was positively associated with i*SD* in corresponding trait manifestations. The fluctuations in neurotic trait manifestations could be related to the reactivity to daily hassles of individuals with high levels of neuroticism (Wrzus et al. 2017). Moreover, higher trait levels in openness were not related to i*SD* in corresponding experiences, but to both i*SD* and diversity in behaviors. This could indicate that openness is characterized by varying in the frequency of open behaviors as well as showing a broad range of diverse types of open behaviors. Trait levels in conscientiousness were not related to variability in the corresponding trait manifestations—a finding that was also observed by Fleeson and Gallagher (2009). It could be that low i*SD* in manifestations of conscientiousness proves functional to remain self-disciplined and persistent across daily life situations.

The present study made a distinction between experiences and behaviors and found that neurotic, extraverted, agreeable, and conscientious experiences and behaviors were interrelated within people. For example, days of more neurotic behaviors were significantly associated with more neurotic experiences. The within-person associations were also evidenced at the between-person level, suggesting that average experiences across the study period were associated with average behaviors. These joint fluctuations of experiences and behaviors indicate congruency in trait manifestations which might reflect a health promoting process (Bono and Vey 2007; Kolanowski et al. 2011). Indeed, previous research on personality and health outcomes in both healthy and clinical populations showed that engagement in personality-incongruent activities was related to more stress and higher heart rate (Bono and Vey 2006). In a clinical study with patients with dementia, matching leisure activities to the patients’ personality significantly improved engagement and cognitive performance compared to patients whose activities were mismatched with their personality (Kolanowski et al. 2011). Congruency in trait manifestations may thus be a mechanism between personality traits and healthy aging outcomes, but future research is needed to investigate this possible pathway. To test whether the coupling between experiences and behaviors varies across age, we conducted additional analyses including age as a moderating variable (Table S6). Results show that age had no moderating effects on the experience-behavior associations, indicating that the coupling remained stable across older adulthood. Future research including a larger sample size is needed to replicate these findings.

The findings for openness did not fit into a coherent picture. Daily open experiences and behaviors were unrelated within and between individuals. One speculative interpretation is that in comparison to open experiences, a higher threshold level is needed to actually manifest open behaviors. This gap between experiences and behaviors underscores the importance of assessing trait manifestations not just as a composite of experiences and behaviors but as separate albeit correlated aspects to better understand how trait content manifests and unfolds in daily life. Future research could address this issue by using different measures of open experiences and behaviors.

### Limitations and future directions

The present study has some limitations that need to be considered. We used different measures to assess personality traits and their manifestations. First, although previous research has shown that the adjective-based TIPI shows good convergent correlations with the BFI (Gosling et al. 2003), the associations between traits and experiences in our study might have been underestimated. Note that despite its potential for ambulatory assessment studies, TIPI was not designed for this type of research. The measure was designed to investigate interindividual differences in the Big Five trait levels. In line with previous work on the manifestations of traits, we adjusted an adjective-based measure of personality traits by changing the time frame of the instruction (from "in general" to "during the last 2 h"). Future research on trait manifestations will benefit from the development of measures that are explicitly tailored to capturing trait manifestations (Horstmann and Ziegler 2020; Ringwald et al. 2020; Zimmermann et al. 2019).

Second, while the separation of affective, cognitive, and behavioral components is distinguishable in theory, a practical distinction seems to be more difficult because a small number of items were not clearly distinctive (e.g., “experienced a lot of stress” for neurotic behavior or “felt cheerful and happy” for extraverted behavior). Although self-reports of trait manifestations are a suitable method due to the subjective nature of the manifestations, further research could benefit from studies including objective indicators of trait manifestations to validate the subjective self-reports.

Third, we assessed experiences in the morning and afternoon, whereas behaviors were only assessed retrospectively in the evening. Further research is needed using different time intervals between measurements of trait manifestations and different statistical approaches to better capture the wide variety of personality manifestations (Neubauer et al. 2019). Finally, future aging research on personality should combine short-term variability in personality manifestations with personality development across older adulthood (Nesselroade 1991).

### Conclusion

The present study offers a fine-grained perspective on how personality traits are expressed in daily life. By distinguishing between trait-related experiences and behaviors, results indicate that trait manifestations were characterized by both relative stability and variability in older age. Several age effects in trait manifestations were found: Age was positively related to neurotic experiences as well as variability in neurotic, extraverted, and open experiences. Furthermore, older adults showed various ways how experiences and behaviors jointly unfold in daily life. These findings contribute to the growing literature on within-person variability in different domains of individual functioning and might encourage further research on personality processes to better understand how older adults experience and behave in daily life.

## Supplementary Information

Supplementary file1 (DOCX 140 kb)

Supplementary file1 (DOCX 50 kb)

## Data Availability

On the Open Science Framework page at https://osf.io/h2pq5/, we published all data to reproduce reported results.

## Footnotes

1 Previous work using this dataset focused on different variables and research questions. The relevant papers investigated the associations of open and neurotic behaviors with cognitive complaints and engagement (Aschwanden et al. 2019), the interrelations among attachment security, need satisfaction, and psychological adjustment (Martin et al. 2019), how future-time perspective is associated with affect (Katana et al. 2020), and the predictive effects of personality traits and cognitive performance on cognitive complaints and engagement (Hill et al. 2020).

2 We changed “did poorly on an assignment or exam” to “performed a task poorly” and “skipped class, work, or other scheduled activities on a whim” to “skipped work or scheduled activities on a whim”.

3 See Mejía et al. (2014) for a method to disentangle reliability and stability of i*SD.*
