# Supplementary material for: How do personality traits manifest in daily life of older adults?
Source: Eur J Ageing. 2021 Feb 17;19(1):131–42. doi: 10.1007/s10433-020-00598-z (PMC8881547; doi:10.1007/s10433-020-00598-z)
Supplement: Supplementary file 1 — Supplementary file1 (DOCX 140 kb) [file 10433_2020_598_MOESM1_ESM.docx]

*Supplementary Figure 1.* A selection of individual trajectories of manifestations of neurotic experiences across 10 days. Individuals in the left panel were characterized by low variability, and individuals in the right panel were characterized by high variability.

Score

| Score  Measurement point | Measurement point |
| --- | --- |


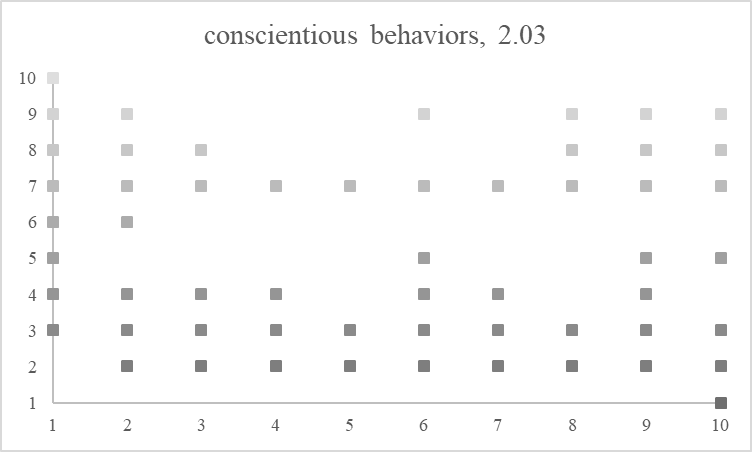

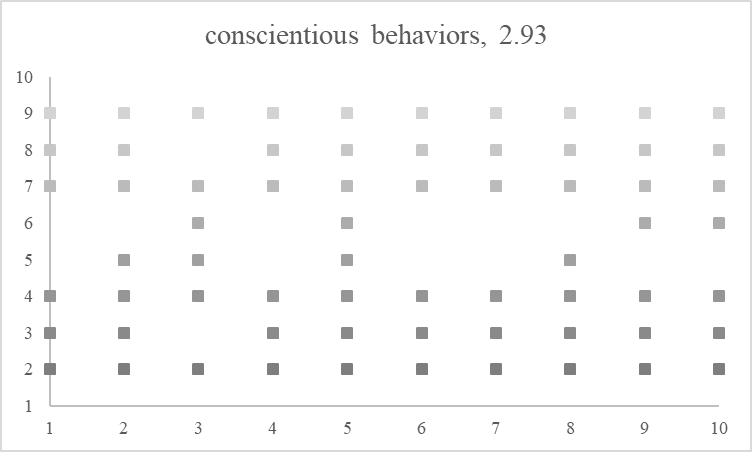

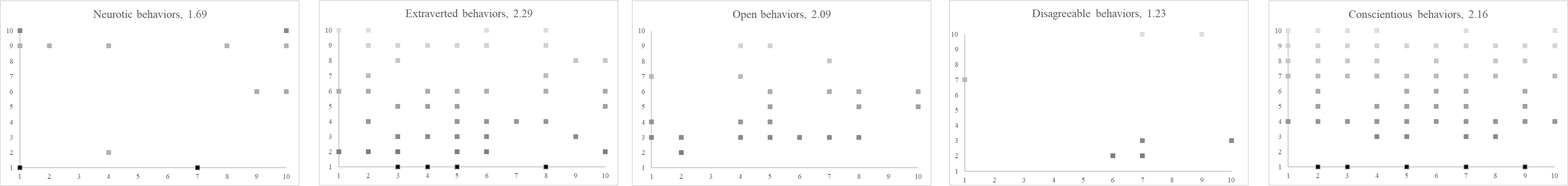


Behavior Behavior

Day Day Day Day Day


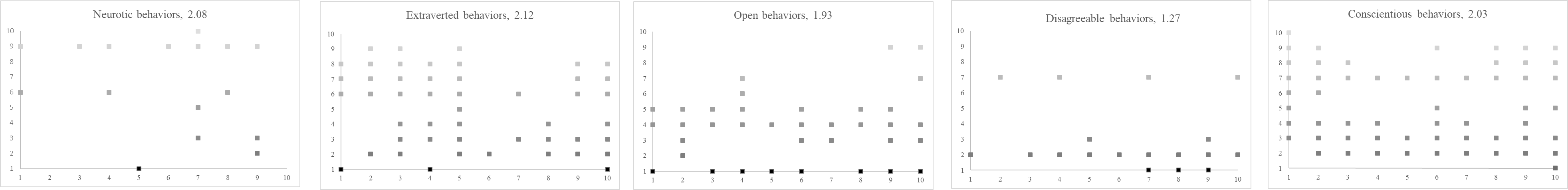


Day Day Day Day Day

*Supplementary Figure 2.* Manifestations of trait-related behaviors across 10 days. Each square represents when a particular behavior (see Supplementary Table 1 in online materials for items) was reported. Graphs at the upper part belong to one individual, graphs at the lower part belong to another individual. Scores represent an individual’s diversity score (Shannon, 1948), calculated with Chao and Shen’s (2003) estimation procedure.
