# Supplementary material for: How do personality traits manifest in daily life of older adults?
Source: Eur J Ageing. 2021 Feb 17;19(1):131–42. doi: 10.1007/s10433-020-00598-z (PMC8881547; doi:10.1007/s10433-020-00598-z)
Supplement: Supplementary file 2 — Supplementary file1 (DOCX 50 kb) [file 10433_2020_598_MOESM2_ESM.docx]

# **Supplemental Materials**

**Appendix S1**

*Description of the Procedure to Assess Diversity of Behaviors*

To assess diversity of behaviors, we used Shannon’s (1948) entropy index following previous research (Ram, Conroy, Pincus, Hyde, & Molloy, 2012; Zhaoyang et al., 2018). Specifically, diversity was calculated as

$behavior diversity = -\left( \frac{1}{ln(m)} \right)\sum_{j=1}^{m} p_{\mathrm{ij}}\mathrm{lnp}_{\mathrm{ij}}$

where *m* is the number of possible behaviors for each Big Five trait (e.g., *m* = 10 behaviors for each Big Five trait) and *p_ij_* is the proportion of individual *i*’s total manifested behaviors with behavior *j* across 10 days. However, Shannon’s entropy index is known to be biased by undersampling (i.e., missings), hereby hindering a correct assessment of behavior diversity (Beck & Schwanghart, 2010; Chao & Shen, 2003). To address this issue, we applied Chao and Shen’s (2003) estimation procedure using the *entropy* package in R (Hausser & Strimmer, 2015). This corrects a sum of measurements for missing behaviors (i.e., the probability for each behavior to be present in the sample) and applies the concept of sample coverage, which is used to properly estimate the relative abundances of behaviors discovered in the sample. As a result, rarely occurring behaviors are weighted more strongly than frequently occurring behaviors in the sample. Without adjustment of Shannon’s entropy index, scores can range from 0 (only one behavior of a Big Five trait was manifested indicating no diversity) to 1 (equal number of behaviors of a Big Five trait were manifested indicating complete diversity). With adjustment, scores can exceed 1. Higher scores indicated more behavior diversity.

**References**

Beck, J., & Schwanghart, W. (2010). Comparing measures of species diversity from incomplete inventories: An update. *Methods in Ecology and Evolution,* *1,* 38–44. https://doi.org/10.1111/j.2041-210X.2009.00003.x

Chao, A., & Shen, T.-J. (2003). Nonparametric estimation of Shannon’s index of diversity when there are unseen species in sample. *Environmental and Ecological Statistics,* *10,* 429–443. https://doi.org/10.1023/A:1026096204727

Hausser, J., & Strimmer, K. (2015). Package "entropy". R package version 1.2.1 http://strimmerlab.org/software/entropy/

Ram, N., Conroy, D., Pincus, A., Hyde, A., & Molloy, L. (2012). Tethering theory to method: Using measures of intraindividual variability to operationalize individuals’ dynamic characteristics. In G. Hancock & J. Harring (Eds.), *Advances in Longitudinal Methods in the Social and Behavioral Sciences* (pp. 81–110). New York, NY: Routledge.

Shannon, C. E. (1948). A mathematical theory of communication. *Bell System Technical Journal, 27,* 379–423. https://doi.org/10.1002/j.1538-7305.1948.tb01338.x

Zhaoyang, R., Sliwinski, M. J., Martire, L. M., & Smyth, J. M. (2018). Age differences in adults’ daily social interactions: An ecological momentary assessment study. *Psychology and Aging, 33,* 607–618. https://doi.org/10.1037/pag0000242

**Appendix S2**

*Full Description of Personality Trait Manifestation*

Descriptive statistics of trait-related experiences are shown in Table 1. Correlations between all variables are shown in Supplementary Table 2. The relative amount of between-person variance for experiences ranged from .54 (open experiences) to .67 (neurotic and conscientious experiences). That is, 54% to 67% of the total variance related to differences between persons. The average daily i*M* ranged from 1.12 (neurotic experiences) to 4.88 (conscientious experiences), suggesting that individuals’ means differed among the Big Five traits. Likewise, the average i*SD* scores ranged from 0.53 (neurotic and conscientious experiences) to 0.80 (extraverted experiences) and showed interindividual differences in the amount of i*SD*. That is, some individuals consistently reported the same experience scores across 10 days (i*SD_min_* = 0), while others varied widely across 10 days (i*SD_max_* = 2.83). Supplementary Figure 1 illustrates a selection of individual trajectories of i*SD* in neurotic experiences. Trajectories in the left panel in Figure 1 represent individuals who reported relatively similar scores of neurotic experiences over the repeated assessments (i*SD* score range = 0-0.87), whereas trajectories in the right panel represent people with more variation in neurotic experiences across 10 days (i*SD* score range = 1.04-2.83). In line with our expectation, stability estimates from Week 1 to Week 2 were higher for i*M* than for i*SD* in experiences. Stability estimates for i*M* were higher than .90 for each Big Five trait, while stability estimates for i*SD* ranged from .54 (agreeable experiences) to .82 (extraverted experiences).

Descriptive statistics of trait-related behaviors are shown in Table 2. The relative amount of between-person variance for behaviors was lower than for experiences and ranged between .31 (neurotic behaviors) and .55 (open behaviors), suggesting that there was more variation within persons. Out of a total of 10 possible daily behaviors for each trait, average daily i*M* ranged from 0.70 (disagreeable behaviors) to 6.13 (conscientious behaviors). The i*SD* scores of behaviors ranged from 0.66 (neurotic behaviors) to 1.89 (extraverted behaviors) and showed interindividual differences in the amount of i*SD*. Diversity scores ranged from relatively low diversity (0.93; neurotic behaviors) to relatively high diversity (2.08; extraverted behaviors). This wide range indicated interindividual differences in behavior diversity. While some individuals showed no diversity in their behavior manifestation (Diversity*_min_* = 0), others manifested a variety of different behaviors (Diversity*_max_* = 4.82). Note that 20.6% of the participants had an entropy score of 0 for neurotic behaviors. For disagreeable behaviors, 16.9%, 17.6%, and 25% of the participants yielded scores of 0 for i*M*, i*SD*, and diversity, respectively. Supplementary Figure 2 illustrates a selection of individual trajectories of daily behaviors across 10 days. The week-to-week stability estimates for i*M* ranged between .43 (neurotic behaviors) to .83 (open behaviors), indicating the degree to which individual differences in behaviors were maintained across 10 days. In comparison, stability estimates for i*SD* were relatively low ranging from .24 (open and disagreeable behaviors) to .34 (extraverted behaviors).

| **Table S1**  *Items from the Daily Behavior Questionnaire (Church et al., 2008, p. 1213)* |
| --- |
| **Neurotic behaviors**  1 Felt anxious about work that needed to be done  2 Experienced a lot of stress  3 Acted moody  4 Felt sad  5 Put myself down  6 Complained about a problem I was having  7 Broke down when a problem arose  8 Felt jealous of someone  9 Kept an even mood during some difficulty^a^  10 Gave in to a bad habit when I was nervous  **Extraverted behaviors**  1 Talked a lot  2 Hugged someone  3 Went out to socialize  4 Chatted with strangers  5 Took the lead in organizing a project or activity  6 Expressed my own opinion  7 Smiled and laughed with others  8 Felt cheerful and happy  9 Mixed well at a social function  10 Introduced myself to someone new  **Openness-to-Experience behaviors**  1 Enjoyed some art  2 Read a play or novel  3 Thought about my emotional reactions to something  4 Experienced some intense feelings  5 Listened to or read with interest a news story about another country  6 Discussed politics  7 Listened with interest to someone whose values or beliefs differed from mine  8 Tried out a new activity for the sake of doing something different  9 Discussed an issue from all points of view  10 Read poetry  **Disagreeable behaviors**  1 Said/did something to hurt someone’s feelings^a^  2 Criticized someone^a^  3 Made a decision without consulting the others involved^a^  4 Yelled at someone^a^  5 Accused someone of talking behind my back^a^  6 Got into an argument^a^  7 Had doubts about someone’s honesty^a^  8 Felt someone betrayed my trust^a^  9 Got what I wanted by withholding the truth from someone^a^  10 Made a rude comment about a person passing^a^  **Conscientious behaviors**  1 Checked out every detail on a task I completed  2 Put my clothes neatly away  3 Did not put away my things when I finished a project or an activity^a^  4 Did poorly on an assignment or exam^a,b^  5 Did an important task well  6 Finished a task on time  7 Forgot about an appointment^a^  8 Finished everything I planned to do today  9 Skipped class, work, or other scheduled activities on a whim^a,b^  10 Reflected on the consequences of an action before going ahead with something  *Note.* ^a^ inverted behaviors; ^b^ In the RHYTHM study, these items were slightly modified to older adults’ daily use. Modified items are “Performed a task poorly” and “Skipped work or scheduled activities on a whim”. |

| **Table S2**  *Pearson Correlations among Intraindividual Mean, Intraindividual Variability, and Diversity of Trait-Related Experiences and Behaviors* | | | | | | | | | | | | | | | | | | | | | | | | | | | | | | | | | | | | | | | | | | | | | | | | | | | |
| --- | --- | --- | --- | --- | --- | --- | --- | --- | --- | --- | --- | --- | --- | --- | --- | --- | --- | --- | --- | --- | --- | --- | --- | --- | --- | --- | --- | --- | --- | --- | --- | --- | --- | --- | --- | --- | --- | --- | --- | --- | --- | --- | --- | --- | --- | --- | --- | --- | --- | --- | --- |
|  | Intraindividual Mean of Experiences (i*M*) | | | | | | | | | | Intraindividual Variability of Experiences (i*SD*) | | | | | | | | | Intraindividual Mean of Behaviors (i*M*) | | | | | | | | | | | Intraindividual Variability of Behaviors (i*SD*) | | | | | | | | | | | Diversity of Behaviors | | | | | | | | | |
|  | 1. N | 2. E | | 3. O | | 4. A | | 5. C | | 6. N | | | 7. E | | 8. O | | 9. A | | 10. C | | | 11. N | | 12. E | | 13. O | | 14. D^a^ | | 15. C | | | 16. N | | 17. E | | 18. O | | 19. D^a^ | | 20. C | | 21. N | | 22. E | | 23. O | | 24. D^a^ | | 25. C |
| 1 | - |  |  | |  | |  | |  | | |  | |  | |  | |  | | |  | |  | |  | |  | |  | | |  | |  | |  | |  | |  | |  | |  | |  | |  | |  | |
| 2 | -.37*** | - |  | |  | |  | |  | | |  | |  | |  | |  | | |  | |  | |  | |  | |  | | |  | |  | |  | |  | |  | |  | |  | |  | |  | |  | |
| 3 | .08 | -.14 | - | |  | |  | |  | | |  | |  | |  | |  | | |  | |  | |  | |  | |  | | |  | |  | |  | |  | |  | |  | |  | |  | |  | |  | |
| 4 | -.59*** | .36*** | -.12 | | - | |  | |  | | |  | |  | |  | |  | | |  | |  | |  | |  | |  | | |  | |  | |  | |  | |  | |  | |  | |  | |  | |  | |
| 5 | -.64*** | .33*** | -.13 | | .47*** | | - | |  | | |  | |  | |  | |  | | |  | |  | |  | |  | |  | | |  | |  | |  | |  | |  | |  | |  | |  | |  | |  | |
| 6 | .56*** | -.18* | .09 | | -.22** | | -.35** | | - | | |  | |  | |  | |  | | |  | |  | |  | |  | |  | | |  | |  | |  | |  | |  | |  | |  | |  | |  | |  | |
| 7 | .13 | -.20** | .17* | | -.03 | | -.07 | | .50*** | | | - | |  | |  | |  | | |  | |  | |  | |  | |  | | |  | |  | |  | |  | |  | |  | |  | |  | |  | |  | |
| 8 | .10 | -.15 | -.11 | | -.13 | | -.07 | | .26** | | | .40*** | | - | |  | |  | | |  | |  | |  | |  | |  | | |  | |  | |  | |  | |  | |  | |  | |  | |  | |  | |
| 9 | .39*** | -.06 | .26** | | -.47*** | | -.18* | | .60*** | | | .46*** | | .25* | | - | |  | | |  | |  | |  | |  | |  | | |  | |  | |  | |  | |  | |  | |  | |  | |  | |  | |
| 10 | .39*** | -.12 | .24** | | -.22** | | -.54*** | | .55*** | | | .43*** | | .17* | | .52*** | | - | | |  | |  | |  | |  | |  | | |  | |  | |  | |  | |  | |  | |  | |  | |  | |  | |
| 11 | .40*** | -.11 | .03 | | .32*** | | -.19* | | .24** | | | .14 | | .03 | | .28*** | | .20** | | | - | |  | |  | |  | |  | | |  | |  | |  | |  | |  | |  | |  | |  | |  | |  | |
| 12 | -.25** | .38*** | -.07 | | .31*** | | .23** | | -.26** | | | -.25*** | | -.22** | | -.23** | | -.19* | | | -.02 | | - | |  | |  | |  | | |  | |  | |  | |  | |  | |  | |  | |  | |  | |  | |
| 13 | -.06 | .07 | -.15 | | .16 | | .04 | | -.07 | | | .03 | | -.13 | | -.16 | | -.03 | | | .12 | | .45*** | | - | |  | |  | | |  | |  | |  | |  | |  | |  | |  | |  | |  | |  | |
| 14 | .23** | -.04 | .05 | | -.19* | | -.05 | | .22** | | | .18* | | .09 | | .18* | | .10 | | | .54** | | .17* | | .33** | | - | |  | | |  | |  | |  | |  | |  | |  | |  | |  | |  | |  | |
| 15 | -.32*** | .24** | -.14 | | .25** | | .38*** | | -.22** | | | -.12 | | -.17* | | -.28*** | | -.24** | | | -.06 | | .35*** | | .37*** | | .07 | | - | | |  | |  | |  | |  | |  | |  | |  | |  | |  | |  | |
| 16 | .35** | -.06 | -.01 | | -.31*** | | -.13 | | .24** | | | .12 | | .08 | | .23** | | .10 | | | .70*** | | .03 | | .14 | | .57*** | | .07 | | | - | |  | |  | |  | |  | |  | |  | |  | |  | |  | |
| 17 | -.05 | .11 | -.15 | | .09 | | .00 | | -.12 | | | .02 | | -.03 | | -.09 | | -.02 | | | .13 | | .28** | | .20* | | .10 | | .06 | | | .09 | | - | |  | |  | |  | |  | |  | |  | |  | |  | |
| 18 | -.01 | .09 | -.10 | | .12 | | .15 | | -.09 | | | .03 | | -.17* | | -.09 | | -.15 | | | .01 | | .28** | | .33*** | | .11 | | .17 | | | -.04 | | .40*** | | - | |  | |  | |  | |  | |  | |  | |  | |
| 19 | .21* | -.10 | .06 | | -.19* | | .01 | | .15 | | | .15 | | .19* | | .15 | | .04 | | | .45*** | | .16 | | .29** | | .73*** | | .06 | | | .46*** | | .37*** | | .33*** | | - | |  | |  | |  | |  | |  | |  | |
| 20 | .03 | .00 | -.12 | | -.18* | | .01 | | -.09 | | | .06 | | .00 | | .04 | | -.02 | | | .26** | | .21* | | .21* | | .28** | | .20* | | | .37*** | | .19* | | .25** | | .33*** | | - | |  | |  | |  | |  | |  | |
| 21 | .34*** | -.19* | -.05 | | -.31*** | | -.17 | | .32*** | | | .22** | | .07 | | .29*** | | .21* | | | .67*** | | -.03 | | .16 | | -.55*** | | .08 | | | .82*** | | .17* | | -.02 | | .48*** | | .24** | | - | |  | |  | |  | |  | |
| 22 | .01 | .10 | -.14 | | .11 | | -.12 | | .05 | | | .07 | | -.19* | | -.06 | | .15 | | | .13 | | .45*** | | .35*** | | .21** | | .17* | | | .12 | | .53*** | | .35*** | | .31*** | | .23** | | -.03 | | - | |  | |  | |  | |
| 23 | .04 | .15 | -.11 | | .05 | | -.08 | | -.02 | | | .02 | | -.09 | | -.05 | | .09 | | | .14 | | .25** | | .35*** | | .24** | | .07 | | | .12 | | .32*** | | .40*** | | .23* | | .18* | | .16 | | .35*** | | - | |  | |  | |
| 24 | .09 | -.10 | -.17 | | .08 | | -.25** | | .40*** | | | .33*** | | .31*** | | .09 | | .07 | | | -.21* | | -.20* | | 02 | | -.30** | | -.13 | | | -.30*** | | .07 | | -.10 | | -.16** | | -.24** | | .55*** | | .21** | | .24** | | - | |  | |
| 25 | -.12 | .13 | -.19* | | .10 | | .12 | | -.10 | | | -.06* | | -.11 | | -20* | | -.08 | | | .07 | | .29*** | | .36*** | | .24** | | .75*** | | | .21* | | .20* | | .20* | | .24** | | .51*** | | .08 | | .17 | | .07 | | -.13 | | - | |
| *Note.* *N* = 136 participants, observations = 2,504 – 2,687; i*SD*: intraindividual standard deviation; ^a^ the Daily Behavior Checklist assessed disagreeable instead of agreeable behaviors (Church et al., 2008).  **p* < .05,***p* < .01,****p* < .001. | | | | | | | | | | | | | | | | | | | | | | | | | | | | | | | | | | | | | | | | | | | | | | | | | | | |

| **Table S3**  *Partial Correlations Between Traits and Trait-Related Experiences, Controlling for Age, Gender, Health, and Education* | | | | | | | | | | | | |
| --- | --- | --- | --- | --- | --- | --- | --- | --- | --- | --- | --- | --- |
|  | Intraindividual mean (i*M)* | | | | | Intraindividual variability (i*SD*) | | | | | | |
| Traits | N | E | O | A | C | | N | E | O | A | C |  |
| Neuroticism | **.51***** | -.29*** | -.07 | -.27**** | -.24** | | **.18*** | .04 | .01 | .05 | .06 |  |
| Extraversion | -.18*** | **.47***** | .06 | .26** | .16 | | .08 | .**09** | .03 | .14 | .12 |  |
| Openness | -.23* | .05 | **-.11** | .19* | .27*** | | -.06 | .21 | **.03** | .02 | -.02 |  |
| Agreeableness | -.34*** | .21** | -.05 | **.39***** | .21* | | -.22** | -.16 | -.16 | **-.22**** | -.16 |  |
| Conscientiousness | -.31*** | .16 | .01 | .30*** | **.50***** | | -.20** | -.04 | -.07 | -.05 | **-.10** |  |
| *Note. N* = 136 participants; correlations between traits and the corresponding experiences are printed in bold face.  **p* < .05, ***p* < .01, ****p* < .001. | | | | | | | | | | | | |

| **Table S4**  *Partial Correlations Between Traits and Trait-Related Behaviors, Controlling for Age, Gender, Health, and Education* | | | | | | | | | | | | | | | | | |
| --- | --- | --- | --- | --- | --- | --- | --- | --- | --- | --- | --- | --- | --- | --- | --- | --- | --- |
|  | Intraindividual mean (i*M*) | | | | | Intraindividual variability (i*SD*) | | | | | | | Diversity | | | | |
| Traits | N | E | O | D^a^ | C | | N | E | O | D^a^ | C | N | | E | O | D^a^ | C |
| N | **.38***** | -.07 | .12 | .26** | -.08 | | **.30***** | .06 | .14 | .31*** | .24* | **.29***** | | .06 | .03 | -.04 | .13 |
| E | -.04 | .**14** | .07 | .11 | .01 | | .00 | **.12** | .08 | .07 | -.10 | -.08 | | **.18*** | .20* | .11 | .04 |
| O | -.04 | .11 | **.26***** | .12 | .12 | | .12 | .07 | **.28**** | .24 | .09 | .07 | | .16 | **.18** | .15 | .11 |
| A | -.24** | .15 | -.05 | **-.23**** | .17 | | -.18 | -.12 | .02 | **-.33***** | -.07 | -.24** | | -.02 | -.05 | **-.04** | -.03 |
| C | -.25** | .10 | .06 | -.07 | .**27***** | | -.11 | -.05 | .03 | -.10 | **-.20*** | -.08 | | -.03 | -.00 | .08 | **.00** |
| *Note. N* = 136 participants; correlations between traits and the corresponding behaviors are printed in bold face; ^a^ the Daily Behavior Checklist assessed disagreeable instead of agreeable behaviors (Church et al., 2008).  * *p* < .05, ** *p* < .01, *** *p* < .001. | | | | | | | | | | | | | | | | | |

| **Table S5**  *Fixed Effects of Multilevel Modeling of Trait-Related Behaviors on Corresponding Experiences with Covariates* | | | | | | | | | | | | | | | | | | | |
| --- | --- | --- | --- | --- | --- | --- | --- | --- | --- | --- | --- | --- | --- | --- | --- | --- | --- | --- | --- |
|  | Experiences | | | | | | | | | | | | | | | | | | |
|  | Neuroticism | | | Extraversion | | | | Openness | | | | Agreeableness^a^ | | | Conscientiousness | | | | |
| Variable | Estimate | | CI 95 % | | Estimate | | CI 95 % | | Estimate | CI 95 % | Estimate | | | CI 95 % | | Estimate | | CI 95 % |  |
| Intercept | 1.10***  (.39) | [.34, 1.86] | | | 2.25***  (.42) | | [1.23, 3.27] | | 3.82***  (.29) | [3.13, 4.51] | 4.71*** (.24) | | | [4.07, 5.35] | | 2.71***  (.52) | | [1.69, 3.72] |  |
| Between | .67***  (.13) | | [.41, .92] | | .23***  (.06) | | [.14, .35] | | -.03  (.05) | [-.13, .07] | -.29*  (.13) | | | [-.53, -.02] | | .26***  (.06) | | [.14, .38] |  |
| Within | .09***  (.02) | | [.05, .13] | | .05***  (.01) | | [.03, .06] | | .02  (.01) | [-.01, .04] | -.07**  (.02) | | | [-.11, -.02] | | .04***  (.01) | | [.02, .05] |  |
| Time | -.01*  (.00) | | [-.01, .00] | | .01  (.00) | | [.00, .01] | | .00  (.00) | [-.01, .00] | .00  (.00) | | | [.00, .01] | | .00*  (.00) | | [.00, .01] |  |
| Age | .02  (.01) | | [.00, .05] | | -.01  (.18) | | [-.04, .02] | | .00  (.01) | [-.02, .02] | .01  (.01) | | | [-.01, .03] | | -.01  (.01) | | [-.03, .02] |  |
| Gender^b^ | -.06  (.15) | | [-.35, .24] | | -.01  (.18) | | [-.40, .32] | | -.04  (.15) | [-.31, .24] | | | .06  (.13) | [-.20, .31] | | .12  (.14) | | [-.16, .40] |  |
| Health | -.30**  (.10) | | [-.51, -.10] | | .39**  (.13) | | [.14, .64] | | -.11  (.10) | [-.31, .08] | .25**  (.09) | | | [.07, .43] | | .26**  (.10) | | [.06, .45] |  |
| Education^c^ |  | |  | |  | |  | |  |  |  | | |  | |  | |  |  |
| 2 | 1.24**  (.40) | | [-2.02, -.45] | | .60  (.51) | [-.39, 1.59] | | | -.18  (.38) | [-.92, .56] | .28  (.36) | | | [-.42, .98] | | .72  (.39) | [-.05, 1.49] | |  |
| 3 | .30  (.52) | | [1.30, .71] | | -.30  (.65) | [-1.58, .97] | | | -.39  (.49) | [-1.34, .55] | .18  (.46) | | | [-.72, 1.08] | | -.06  (.51) | [-1.05, .93] | |  |
| 4 | -.76*  (.38) | | [-1.51 -.01] | | .41  (.49) | [-.54, 1.36] | | | -.36  (.37) | [-1.08, .36] | .37  (.34) | | | [-.30, 1.04] | | .57  (.38) | [-.17, 1.31] | |  |
| 5 | .58  (.40) | | [-1.36, .20] | | -.13  (.51) | [-1.12, .86] | | | -.03  (.38) | [-.77, .71] | .01  (.36) | | | [-.69, .70] | | .25  (.40) | [-.52, 1.02] | |  |
| 6 | -.72  (.38) | | [-1.46, .03] | | .17  (.48) | [-.77, 1.12] | | | -.14  (.36) | [-.84, .57] | .25  (.34) | | | [-.41, .92] | | .51  (.38) | [-.23, 1.24] | |  |
| *Note.* *N* = 136 participants, observations = 2,493; estimates are unstandardized multilevel regression coefficients with standard errors in parentheses; between: between-person version of the independent variable; within: within-person version of the independent variable; CI: confidence interval; ^a^ the independent variable was disagreeable behaviors as assessed with the Daily Behavior Checklist (Church et al., 2008); ^b^ 0: female, 1: male; ^c^ reference category: secondary school with lower school track, 2: secondary school with higher school track, 3: secondary school with Matura graduation, 4: university of applied sciences, 5: university, 6: others.  **p* < .05, ***p* < .01, ****p* < .001. | | | | | | | | | | | | | | | | | | | |

| **Table S6**  *Fixed Effects of Multilevel Modeling of Trait-Related Behaviors on Corresponding Experiences with Age Moderation* | | | | | | | | | | | | | | |
| --- | --- | --- | --- | --- | --- | --- | --- | --- | --- | --- | --- | --- | --- | --- |
|  | Experiences | | | | | | | | | | | | | |
|  | Neuroticism | | | Extraversion | | | Openness | | | Agreeableness^a^ | | | Conscientiousness | |
| Behavior | Estimate | CI 95 % | Estimate | | CI 95 % | Estimate | | CI 95 % | Estimate | | CI 95 % | Estimate | | CI 95 % |
| Intercept | .33  (.18) | [-.02, .68] | 2.28 ***  (.26) | | [1.77, 2.79] | 3.67 ***  (.15) | | [3.38, 3.96] | 5.00 ***  (.09) | | [4.81, 5.18] | 3.10 ***  (.39) | | [2.33, 3.85] |
| Between | .69 ***  (.13) | [.42 .94] | .29 ***  (.06) | | [.16, .40] | -.04  (.05) | | [-.13, .04] | -.34 **  (.13) | | [-.59, -.08] | .29 ***  (.06) | | [.16, .40] |
| Within | .09 ***  (.02) | [.05, .12] | .05 ***  (.01) | | [.03, .06] | .02  (.01) | | [-.00, .03] | -.07 **  (.02) | | [-.11, -.02] | .04 ***  (.01) | | [.01, .05] |
| Age | -.01  (.03) | [-.05, .04] | -.01  (.04) | | [-.07, .05] | .02  (.02) | | [-.02, .06] | -.01  (.01) | | [-.03, .02] | -.05  (.05) | | [-.16, .05] |
| Time | -.01  (.00) | [-.01, -.00] | .01  (.00) | | [-.00, .01] | -.00  (.00) | | [.00, .00] | .00  (.00) | | [.00, .00] | .00  (.00) | | [.00, .00] |
| Between*Age | .03  (.02) | [-.01, .06] | .00  (.01) | | [.00, .00] | -.01  (.01) | | [-.02, .01] | .03  (.02) | | [-.01, .05] | .01  (.01) | | [-.01, .02] |
| Within*Age | .00  (.00) | [.00, .00] | .00  (.00) | | [.00, .00] | .00  (.00) | | [.00, .00] | .00  (.00) | | [.00, .00] | .00  (.00) | | [.00, .00] |
| *Note. N* = 136 participants, observations = 2,493; estimates are unstandardized multilevel regression coefficients with standard errors in parentheses; between: between-person version of the independent variable; within: within-person version of the independent variable; CI: confidence interval; ^a^ the independent variable was disagreeable behaviors as assessed with the Daily Behavior Checklist (Church et al., 2008).  **p* < .05, ***p* < .005, ****p* < .001. | | | | | | | | | | | | | | |
